# Adding New Pieces to the Puzzle of Karyotype Evolution in *Harttia* (Siluriformes, Loricariidae): Investigation of Amazonian Species

**DOI:** 10.3390/biology10090922

**Published:** 2021-09-16

**Authors:** Francisco de M. C. Sassi, Orlando Moreira-Filho, Geize A. Deon, Alexandr Sember, Luiz A. C. Bertollo, Thomas Liehr, Vanessa C. S. Oliveira, Patrik F. Viana, Eliana Feldberg, Marcelo R. Vicari, Marcelo de B. Cioffi

**Affiliations:** 1 Laboratório de Citogenética de Peixes, Departamento de Genética e Evolução, Universidade Federal de São Carlos, São Carlos 13565-905, SP, Brazil; fmcsassi@estudante.ufscar.br (F.d.M.C.S.); omfilho@ufscar.br (O.M.-F.); gdeon@estudante.ufscar.br (G.A.D.); bertollo@ufscar.br (L.A.C.B.); vcsoliveira@estudante.ufscar.br (V.C.S.O.); mbcioffi@ufscar.br (M.d.B.C.); 2 Laboratório de Biologia Cromossômica, Estrutural e Função, Departamento de Biologia Estrutural, Molecular e Genética, Universidade Estadual de Ponta Grossa, Ponta Grossa 84030-900, PR, Brazil; vicarimr@pq.cnpq.br; 3 Laboratory of Fish Genetics, Institute of Animal Physiology and Genetics, Czech Academy of Sciences, Rumburská 89, 277-21 Liběchov, Czech Republic; sember@iapg.cas.cz; 4 Institute of Human Genetics, University Hospital Jena, Friedrich Schiller University, 07747 Jena, Germany; 5 Laboratório de Genética Animal, Coordenação de Biodiversidade, Instituto Nacional de Pesquisas da Amazônia, Manaus 69067-375, AM, Brazil; patrik.biologia@gmail.com (P.F.V.); feldberg@inpa.gov.br (E.F.)

**Keywords:** chromosomes, comparative genomic hybridization (CGH), repetitive DNA, sex chromosomes

## Abstract

**Simple Summary:**

Fishes represent a useful model for evolutionary studies, given their diversity of species and habitats. In this study, we investigate the chromosomes of three unexplored *Harttia* fish species from the Amazonian region and compare the obtained data with previous analyses. Our data reveal that both *Harttia dissidens* and *Harttia* sp. 3 exhibit the same number of chromosomes in their cells (54), but that they differ in the karyotype organization. *Harttia guianensis* possesses 58 chromosomes, being thus the first representative from north Brazil to present this feature for both sexes. Although otherwise rather common in *Harttia* species, we observed no chromosomal differences between sexes in all but one species. Namely in *Harttia* sp. 3, we revealed signs of initial differentiation between homologues of one chromosome pair in males but not in females. Altogether, our data bring new evidence strengthening the view that *Harttia* spp. represent an informative model for studying patterns of karyotype and sex chromosome dynamics in teleost fishes.

**Abstract:**

A remarkable morphological diversity and karyotype variability can be observed in the Neotropical armored catfish genus *Harttia*. These fishes offer a useful model to explore both the evolution of karyotypes and sex chromosomes, since many species possess male-heterogametic sex chromosome systems and a high rate of karyotype repatterning. Based on the karyotype organization, the chromosomal distribution of several repetitive DNA classes, and the rough estimates of genomic divergences at the intraspecific and interspecific levels via Comparative Genomic Hybridization, we identified shared diploid chromosome numbers (2n = 54) but different karyotype compositions in *H. dissidens* (20m + 26sm + 8a) and *Harttia* sp. 3 (16m + 18sm + 14st + 6a), and different 2n in *H. guianensis* (2n = 58; 20m + 26sm + 2st + 10a). All species further displayed similar patterns of chromosomal distribution concerning constitutive heterochromatin, 18S ribosomal DNA (rDNA) sites, and most of the surveyed microsatellite motifs. Furthermore, differences in the distribution of 5S rDNA sites and a subset of microsatellite sequences were identified. Heteromorphic sex chromosomes were lacking in *H. dissidens* and *H. guianensis* at the scale of our analysis. However, one single chromosome pair in *Harttia* sp. 3 males presented a remarkable accumulation of male genome-derived probe after CGH, pointing to a tentative region of early sex chromosome differentiation. Thus, our data support already previously outlined evidence that *Harttia* is a vital model for the investigation of teleost karyotype and sex chromosome dynamics.

## 1. Introduction

With more than 35,000 species [1], fishes have the richest biodiversity among vertebrates [2,3]. The evolutionary success of ray-finned and especially teleost fishes seem to be, at least partially, related to a higher plasticity of their genomes when compared to other vertebrates [4,5,6,7], going hand in hand with their propensity towards polyploidization and hybridization events [8,9]. Another factor at play is the wealth of aquatic niches fishes adapted to [2,10,11,12], and the geomorphological parameters affecting the dispersion and populational dynamics in particular species (e.g., [13,14]). In effect, at the level of genome organization, fishes present a wide range of chromosome counts and genome sizes [15,16] and the karyotype dynamics may differ considerably among lineages [17]. Finally, fishes encompass astounding variability in mechanisms of sex determination and differentiation [18,19,20,21,22].

Siluriformes (Teleostei, Ostariophysi) represent a lineage with high diversification, comprising more than 3000 species distributed among 39 families [1]. Inside Siluriformes, the family Loricariidae whose representatives are commonly known as “armored catfishes” (due to ossified plates covering their bodies; [23]) encompasses 1015 recognized species included in more than 100 genera [1]. It is hence the most species-rich siluriform family. These fishes are distributed throughout the Neotropical region, but with the greatest diversity being found from north Costa Rica to south Argentina [24]. Notably, the diversification is associated with huge karyotype variability in Loricariidae, with diploid chromosome numbers (2n) ranging from 36 in *Rineloricaria latirostris* to 74 in *Sturisoma* cf. *nigrirostrum* [25,26,27]. Additionally, several types of sex chromosome systems including standard, derived, and multiple ones have been described for Loricariidae, as exemplified by *Hypostomus* genus with different ZW and XY cases [28,29,30,31], XX/XY, XX/X0, ZZ/ZW, and Z_1_Z_1_Z_2_Z_2_/Z_1_Z_2_W_1_W_2_ in several *Ancistrus* species (reviewed in ref. [32]), and three male-heterogametic systems in the genus *Harttia* (see below). 

In this context, the genus *Harttia* can be recognized as a remarkable model for cytogenetic studies, especially to investigate the evolutionary processes related to sex chromosomes, diversification and karyotype dynamics [33,34,35,36]. *Harttia* harbors 27 recognized species [1,37], of which only 14 have been cytogenetically analyzed to date, along with several other *Harttia* spp. waiting for proper taxonomic description (summarized in [35]). This genus also encompasses the second-largest variation in the 2n among Loricariidae, ranging from 2n = 52 (females)/53 (males) in *Harttia carvalhoi* [33] to 2n = 62 in *H*. *absaberi* and *Harttia* sp. 2 [35,38]. Furthermore, three different male-heterogametic sex chromosome systems are known or have been suggested to exist among *Harttia* species: XX/XY_1_Y_2_ in *H*. *carvalhoi*, *H*. *intermontana*, and *Harttia* sp. 1 [33,35], X_1_X_1_X_2_X_2_/X_1_X_2_Y in *H*. *punctata*, *H*. *villasboas,* and *H*. *duriventris* [34,36], in addition to a putative XX/XY in *H*. *rondoni* [36].

*Harttia kronei* was the first species analyzed cytogenetically in the genus [39] and, for a long time, a single representative of *Harttia* was studied from this perspective in the northern Brazilian region: the species *H*. *punctata* [34]. However, this gap has been progressively closed with our previous studies on *Harttia duriventris*, *H*. *rondoni*, and *H*. *villasboas* [36]. Here, we aim to cytogenetically investigate another three representatives from this region. For this, we applied C-banding, repetitive DNA mapping, and comparative genomic hybridization (CGH) experiments in *H*. *dissidens*, *H*. *guianensis*, and *Harttia* sp. 3. Our results highlight the high chromosomal dynamics within the investigated species and a putative early stage of sex chromosome differentiation, particularly in *Harttia* sp. 3. 

## 2. Materials and Methods

### 2.1. Sampling

**Figure 1:**
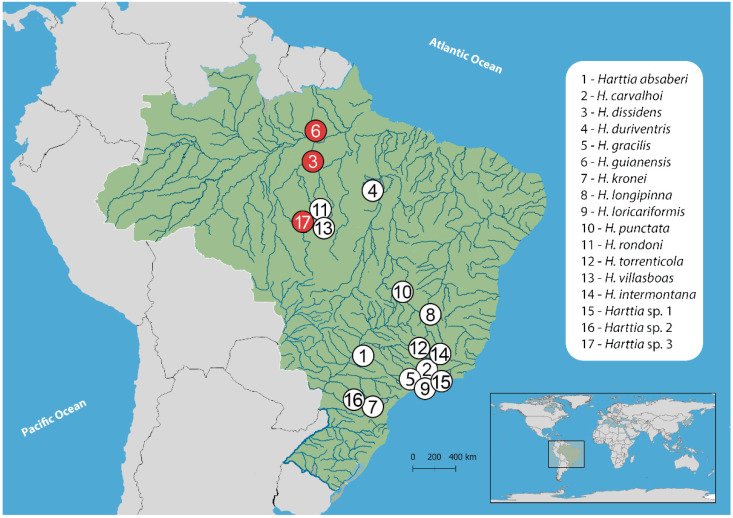
Brazilian territory (green), and the *Harttia* species cytogenetically analyzed in former reports [34,35,36,38] (white circles), including the present study (red circles). Each *Harttia* species is presented by a certain number; the table with the coding system is presented in the white frame on the right.

Three collection sites in the Brazilian Pará state (Figure 1, Table 1) were accessed, allowing the sampling of three species: *Harttia dissidens*, *H. guianensis*, and another yet undescribed taxon herein referred to as *Harttia* sp. 3. 

Sampling was done with the authorization of the environmental agency ICMBIO/SISBIO (License 48628-14) and SISGEN (A96FF09). The species *Harttia dissidens* and *H. guianensis* were identified by Dr. Lúcia Helena Rapp-Py Daniel, curator of the fish collection of the Instituto Nacional de Pesquisa da Amazônia (INPA) and deposited there under the voucher numbers INPA-ICT 059577 and INPA-ICT 059584, respectively. The specimens of *Harttia* sp. 3 were scientifically described by Dr. Oswaldo Takeshi Oyakawa, from the Museu de Zoologia da Universidade de São Paulo (MZUSP). 

### 2.2. Chromosome Obtainment and C-Banding

The living animals were anesthetized with Eugenol before the anterior kidney extraction for the obtainment of mitotic chromosomes following the classical air-drying protocol [40]. Experiments followed the ethical conducts approved by the Ethics Committee on Animal Experimentation of the Universidade Federal de São Carlos (Process number CEUA 1853260315). The detection of constitutive heterochromatin regions followed the C-banding protocol [41]. The C-banding procedure was performed on the slides previously used for the mapping of ribosomal DNAs, to allow a sequential analysis of the same metaphase plates.

### 2.3. Fluorescence In Situ Hybridization (FISH)-Based Experiments 

Ribosomal DNA (rDNA), microsatellite, and the telomeric (TTAGGG)*_n_* sequences were used as probes for chromosomal mapping by FISH. The 5S rDNA probe was prepared according to Pendás et al. [42] from *Hoplias malabaricus* genome, containing a 120-base pair (bp) segment of the 5S rDNA transcribed region and 200 bp of a non-transcribed spacer (NTS). This probe was red-labeled with ATTO550-dUTP using the Nick-Translation mix kit (Jena Bioscience, Jena, Germany). The 18S rDNA probe, containing a 1400 bp-long segment of the transcribed region was also isolated from *H. malabaricus* following Cioffi et al. [43] and green-labeled with AF488-dUTP using the Nick-Translation mix kit (Jena Bioscience). The microsatellites (A)_30_, (CA)_15_, and (GA)_15_ were selected based on their abundance and patterns of chromosome distribution encountered in previous studies in different fish species [36,44], and the corresponding probes were directly labeled with Cy3 (Sigma-Aldrich, Darmstadt, Germany) during their synthesis, as described in [45]. Telomeric sequence (TTAGGG)*_n_* was in situ located using the DAKO Telomere PNA FISH Kit/FITC (DAKO, Glostrup, Denmark). High stringency conditions described in [46] were used in all FISH experiments. As a final step for all fluorescence assays, chromosomes were counterstained with 4′,6-diamidino-2-phenylindole (DAPI), and the slides were mounted in an antifade solution (VECTASHIELD; Vector Laboratories, Burlingame, CA, USA). Two sets of comparative genomic hybridization (CGH) experiments were designed to seek for the occurrence of sex chromosomes, both following the protocol of Sember et al. [47], with adaptations on probe/C_0_t−1 DNA ratio based on previous *Harttia* studies [35,36]. In the first set of experiments, male and female genomic probes were co-hybridized to the male chromosomes (i.e., the expected heterogametic sex), in each species separately, to detect regions with specific or biased hybridization of male probe which might point to regions with sex-specific repetitive DNA accumulation on sex chromosomes. For this experimental scheme, male and female-derived whole-genome DNAs (gDNAs) of all analyzed species were extracted from the liver tissue by the standard phenol-chloroform-isoamyl-alcohol method [48] and labeled with ATTO550-dUTP (male gDNA in red) and AF488-dUTP (female gDNA in green) using the Nick Translation mix kit (Jena Bioscience). To block the excess of shared repetitive sequences, an unlabeled C_0_t-1 DNA, obtained from each species according to Zwick et al. [49] was included in the final probe mixture. For each slide, male and female genomic probe (500 ng each) and 25 µg of female-derived C_0_t−1 DNA from the respective species were co-precipitated in 96% ethanol, and the dry pellets were resuspended in 20 µL of the hybridization buffer containing 50% formamide, 2×SSC, 10% dextran sulfate, and Denhardt’s buffer (pH 7.0). In the second CGH assay, the genomes of *H. dissidens* and *H. guianensis* were compared to one of *H. villasboas*, which possesses an X_1_X_1_X_2_X_2_/X_1_X_2_Y sex chromosome system and whose chromosome preparations were previously obtained by our research group [36]. For this purpose, male-derived gDNAs from *H. dissidens*, *H. guianensis*, and *H. villasboas* were extracted as described above and labeled with fluorochromes emitting green (AF488-dUTP), cyan (ATTO425-dUTP), and red (ATT0550-dUTP) fluorescence, with the Nick Translation mix kit (Jena Bioscience). The final probe mixture was composed of 500 ng of male-derived gDNA of each species and 10 µg of female-derived C_0_t−1 DNA of each species. This probe was then hybridized to the male metaphase plates of *H. villasboas*. 

### 2.4. Image Analysis and Processing

The 2n, karyotype structure, distribution of repetitive DNAs, and CGH results were confirmed by the evaluation of results in at least 30 metaphase spreads per individual. Images were captured using an Olympus BX50 microscope (Olympus Corporation, Ishikawa, Japan), coupled with a CoolSNAP camera, and processed using Image-Pro Plus 4.1 software (Media Cybernetics, Silver Spring, MD, USA). Chromosomes were classified as metacentric (m), submetacentric (sm), subtelocentric (st), or acrocentric (a) according to their arm-ratios [50]. Figures were organized with Adobe Photoshop CC 2020.

## 3. Results

**Figure 2:**
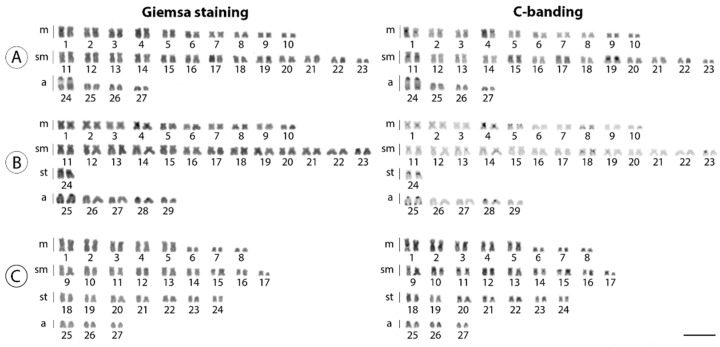
Karyotypes of three *Harttia* species arranged from mitotic metaphases after Giemsa staining, and C-banding. *Harttia dissidens* (**A**), *H. guianensis* (**B**), and *Harttia* sp. 3 (**C**). Scale bar = 10 µm.

**Figure 3:**
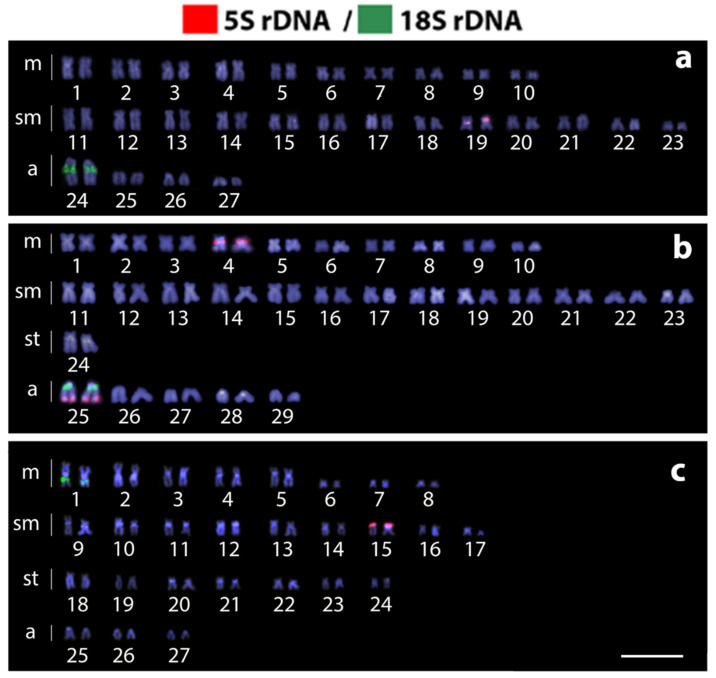
Karyotypes of *Harttia dissidens* (**a**), *H. guianensis* (**b**), and *Harttia* sp. 3 (**c**) after dual-color FISH: 5S (red signals) and 18S (green signals) rDNA. Note the adjacent position of 5S and 18S rDNA signals on chromosome pair No. 25 in *H. guianensis* (**b**). Chromosomes were counterstained with DAPI (blue). Scale bar = 10 µm.

*Harttia dissidens* and *Harttia* sp. 3 exhibited the same 2n = 54 chromosomes but differed in the karyotype composition: 20m + 26sm + 8a and 16m + 18sm + 14st + 6a, respectively. On other hand, *H. guianensis* differed from the other two species both in 2n and karyotype constitution: 2n = 58, and 20m + 26sm + 2st + 10a chromosomes (Figure 2). In *H. dissidens* and *H. guianensis*, constitutive heterochromatin was located preferentially in the pericentromeric and terminal regions of several chromosomes, but with distinct patterns of distribution between the species. In turn, *Harttia* sp. 3 differed by having larger C-positive bands, with signals distributed in the pericentromeric regions of almost all chromosomes (Figure 2). The mapping of the 18S rDNA sequence revealed a single chromosome pair bearing these sites in all three species (pair 24 in *H. dissidens*, pair 25 in *H. guianensis*, and pair 1 in *Harttia* sp. 3). The 5S rDNA cluster was found in a single chromosome pair in *H. dissidens* (pair 19) and *Harttia* sp. 3 (pair 15), and in two chromosome pairs (4 and 25) in *H. guianensis*. It is also notable that both 5S and 18S rDNA sites present a syntenic location on chromosome pair 25 in *H. guianensis* (Figure 3b).

**Figure 4:**
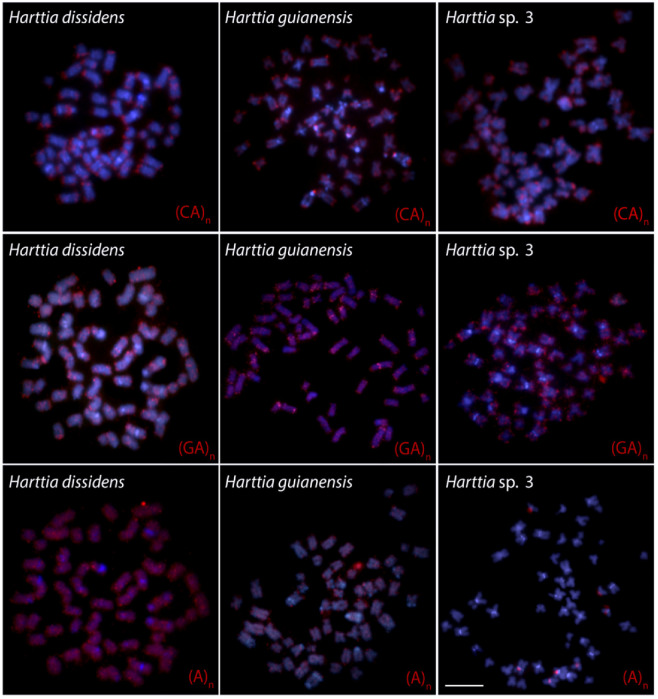
Chromosomal mapping of three microsatellite sequences on *Harttia* mitotic chromosomes. Probes (red signals): (A)_30_, (CA)_15_, and (GA)_15_. Chromosomes were counterstained with DAPI (blue). Scale bar = 10 µm.

**Figure A1:**
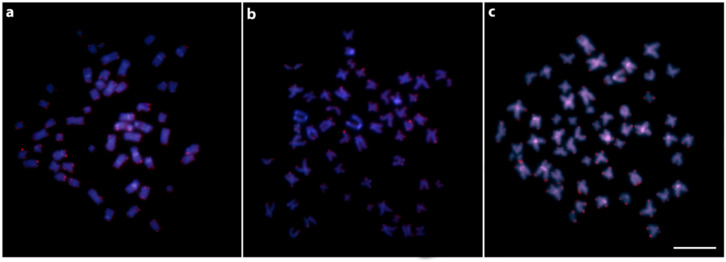
Distribution of the telomeric probe (TTAGGG)*_n_* directly labeled with Cy3-dUTP (red) and hybridized against the DAPI-counterstained (blue) chromosomes of *Harttia dissidens* (**a**), *H. guianensis* (**b**) and *Harttia* sp. 3 (**c**). Bar scale = 10 µm.

The mapping of three microsatellite sequences revealed a similar pattern for (CA)_15_ and (GA)_15_, with accumulation at the terminal regions of all chromosomes. The last studied motif, (A)_30_, presented a dispersed distribution throughout the chromosome complement of *H. dissidens* and *H. guianensis*, while it was accumulated in the pericentromeric regions of few chromosome pairs in *Harttia* sp. 3 (Figure 4). The mapping of the telomeric (TTAGGG)*_n_* sequence revealed only the standard pattern in all the three species, i.e., positive signals at the physical ends of all chromosomes (Figure A1).

**Figure 5:**
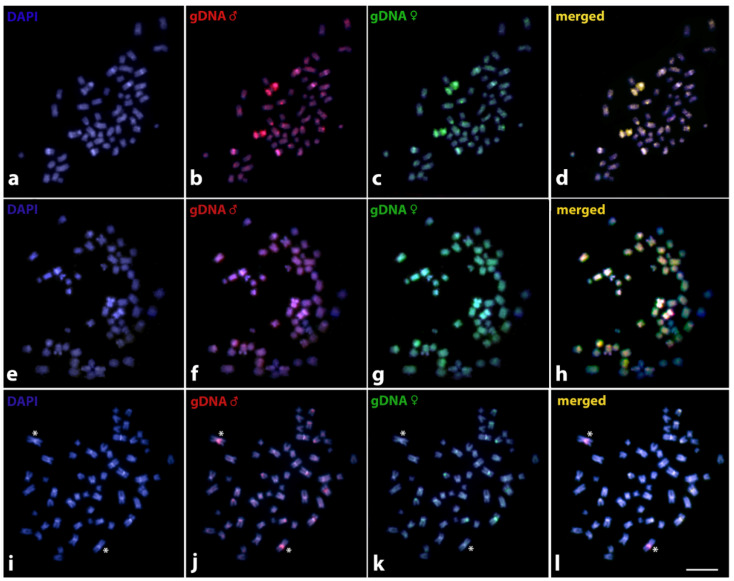
Mitotic chromosome spreads of *Harttia* males after intraspecific (male-to-female) CGH procedure. *Harttia dissidens* (**a**–**d**), *H. guianensis* (**e**–**h**), and *Harttia* sp. 3. (**i**–**l**). First column (**a**,**e**,**i**): DAPI images (blue); second column (**b**,**f**,**j**): hybridization patterns of male gDNA probes; third column (**c**,**g**,**k**): hybridization patterns of female gDNA probes; fourth column (**d**,**h**,**l**): merged images of both genomic probes and DAPI counterstaining. Regions with excessive hybridization of both genomic probes are yellow (i.e., combination of green and red). Asterisks highlight the regions with preferential accumulation of male gDNA probe when compared to patterns of a female gDNA probe hybridization. Scale bar = 10 µm.

The CGH-based experiments between sexes in each of the three analyzed species showed approximately equal binding of both male- and female-specific probes to the vast majority of chromosomes in the male complement, with preferential location to regions with high content of repetitive DNA (yellow signals, i.e., combination of green and red fluorescence). In *Harttia dissidens* and *H. guianensis*, CGH failed to reveal any region with predominant or exclusive hybridization of the male-specific probe, thus we did not observe any signs of potential sex chromosome molecular differentiation at the level detectable by this methodological approach. In *Harttia* sp. 3, however, the male genomic probe predominantly (but not exclusively) marked the pericentromeric region of one large-sized metacentric chromosome pair (Figure 5).

**Figure 6:**
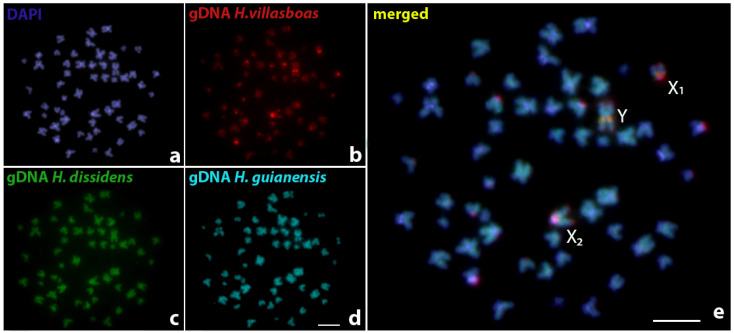
Mitotic metaphase from *Harttia villasboas* male after interspecific comparative genomic hybridization (CGH). Chromosome spreads were probed with male-derived genomic probes from *Harttia villasboas* (red) (**b**), *H. dissidens* (green) (**c**), and *H. guianensis* (light blue) (**d**). The composed image (**e**) contains all three merged genomic probes and DAPI counterstaining (**a**). The genomic regions with shared excessive hybridization of applied male genomic probes are yellowish. Scale bar = 10 µm.

We further performed a second CGH assay with hybridization of the male genomic probes from *Harttia dissidens*, *H. guianensis*, and *H. villasboas* against the chromosomal set of *H. villasboas* (i.e., the species previously reported to have X_1_X_1_X_2_X_2_/X_1_X_2_Y multiple sex chromosome system; see ref. [36]). We found uniform hybridization patterns of both *H. dissidens* and *H. guianensis* probes across the chromosome complement of *H. villasboas* males. The *H. villasboas* male probe seldom predominantly highlighted the regions of high repetitive content, pointing to the fact that the other two compared genomes do not (qualitatively or quantitatively) share this specific portion of *H. villasboas* repetitive DNA classes (Figure 6).

## 4. Discussion

### 4.1. New Pieces into the Chromosomal Evolution of Harttia 

In a previous chromosomal study in *Harttia* species, 2n = 58 was proposed as the likely ancestral 2n for this genus [34]. A few years later, the karyotype diversity among *Harttia* species was described as the result of three different evolutionary pathways: (i) the maintenance of the ancestral 2n = 58 up to today; (ii) the elevation of 2n by centric fissions, and (iii) the 2n reduction by fusions [35]. Here, we add new pieces to this puzzle by analyzing the karyotypes of another three *Harttia* species.

In the most recent phylogenetic analysis of the subfamily Loricariinae [51], the existence of three clades within the *Harttia* genus was proposed. The earliest-diverging clade is composed of *H. guianensis*, *H. tuna*, *H. fluminensis*, and *H. surinamensis* which inhabit waters from the Guiana shield. The two more recently diverging clades display a well-defined and non-overlapping geographical distribution. Their species are found in southeast and south Brazilian regions, which are separated from the northern rivers. Until the present study, cytogenetical investigations only comprised fishes from the latter two clades [34,35,36]. 

The description of the *H. guianensis* karyotype in the present study reinforces the proposal of 2n = 58 as the ancestral 2n for the genus [34]. If we keep in mind that this species is basalmost in the clade, as pointed by phylogenetic reconstructions [51,52], and that it shares the same 2n with other *Harttia* species from other clades, such as *H. gracilis*, *H. longipinna*, and *H. punctata*, such hypothesis is still valid but lacks profound reconstruction analysis. Furthermore, although not a common occurrence, the syntenic organization of at least some sites of both rDNA classes is also considered as a basal karyotype feature for Loricariidae fishes [53]; this is a pattern that we also found in the karyotype of *H. guianensis*, in which 5S and 18S rDNA site share the location on the chromosome pair 25. Interestingly, all three *Harttia* species under study share one 5S rDNA site, which might be hypothetically located on homeologous chromosomes (pairs 19, 4, and 15 in *H. dissidens*, *H. guianensis*, and *Harttia* sp. 3, respectively). The mentioned chromosome pairs only show mild changes in shape and size between species and they differ also regarding the position of an 5S rDNA site (terminal vs. more proximal), which could be explained by paracentric inversions. The second extra 5S rDNA site found in *H. guianensis* only, i.e., the one found in synteny with 18S rDNA cluster on chromosome pair 25 is a karyotype trait that has been so far reported only for two other *Harttia* species: *H. carvalhoi* [34] and *Harttia* sp. 1 [35]. Given the position of *H. guianensis* at the base of *Harttia* phylogeny [51], it seems probable that this second 5S rDNA site has been eliminated during evolution in many *Harttia* species while it was retained in a subset of them. 

The 5S rDNA site has been either entirely eliminated or reduced in copy number of repeats below the resolution of the standard FISH protocol. Based on tracking the location of the 18S rDNA site, we may infer that particularly this linkage group underwent a dynamic evolution (see below). Based on the comparison of 18S rDNA patterns, it is highly probable that the chromosome pair bearing the 18S rDNA site was involved in a fusion event in *H. dissidens* and *Harttia* sp. 3. This may be inferred from the terminal location of 18S rDNA site on a rather middle-sized chromosome pair in *H. guianensis* vs. an interstitial position of 18S rDNA cluster on large-sized chromosomes in the remaining two species. While the presence of a large metacentric pair in *Harttia* sp. 3 supports centric fusion as an underlying mechanism, the large 18S rDNA-bearing chromosome in *H. dissidens* is acrocentric and hence either tandem fusion took place here or the situation was more complex (i.e., involving more consecutive rearrangements). 

For loricariid genomes, it was proposed that the evolutionary breakpoint regions occur adjacent to rDNA sites, promoting double-strand breaks and the reorganization of these sequences in *Ancistrus* [54], *Harttia* [35,36], and *Rineloricaria* [55]. The number of studies showing rDNA sites inside or nearby a predicted fusion points is steadily growing for fishes [56,57,58,59] and a high potential of rDNAs to facilitate chromosome rearrangements has also been shown in mice and humans (e.g., [60,61]). A possible explanation for rDNA instability might be a high transcriptional activity of tandemly arrayed rDNA genes due to the necessity to synthetize high amounts of ribosomes in the cell [62]. Long stretches of transcriptionally active euchromatin might be prone to double-strand breaks [63,64]. Interestingly, mapping of telomeric repeats did not reveal any interstitial telomeric sites (ITSs), which could point to regions of former rearrangements, in our case specifically fusions or inversions; therefore, it seems that the mechanisms of these rearrangements in *Harttia* usually do not preserve these sequences in the fusion points [65,66].

The two species analyzed herein, *H. dissidens* and *Harttia* sp. 3, share the same chromosome count (2n = 54) as was previously only described in *H. rondoni* [36], a species that also inhabits the northern Brazilian region. Indeed, *H. dissidens* occurs in the Tapajós river basin [67], and *H. rondoni* inhabits the Xingu River basin [68]. The range of distribution of *Harttia* sp. 3 cannot be inferred from the current data. This suspected new *Harttia* species was collected in a small rocky-bottom river located on the edge of Serra do Cachimbo, 700 m highlands dividing the Tapajós and Xingu basins. Therefore, it is not yet known whether it flows into the Tapajós River basin or the Xingu River basin. 

**Figure 7:**
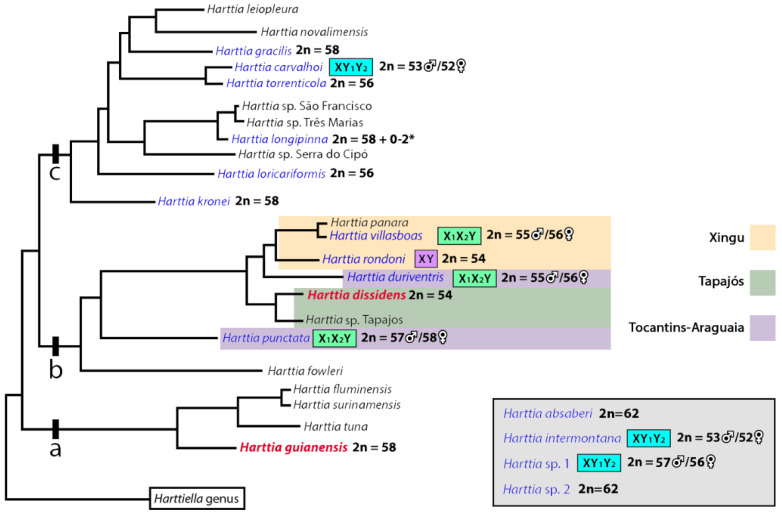
Updated schematic representation of the phylogenetic relationships between *Harttia* species (modified from our formerly published scheme—see ref. [36], based on the molecular data (see ref. [52]), and with plotted cytogenetic characteristics based on previous cytogenetic studies (blue), and the present study (red). Differentiated sex chromosomes are indicated in boxes as follows: X_1_X_2_Y (green box), XY_1_Y_2_ (light blue box), and XY (pink box). Species listed in the gray box (placed at the bottom-right corner) were cytogenetically investigated but their position in the phylogenetic reconstruction needs yet to be determined. River basins where norther *Harttia* species occur are highlighted as follows: Xingu (yellow), Tapajós (green), and Tocantins-Araguaia (purple). Asterisks correspond to extra B chromosomes that can be found in *H. longipinna* karyotype [82].

Although the karyotypes of *H. dissidens*, *H. rondoni*, and *Harttia* sp. 3 share 2n equal to 54 chromosomes, notable differences can be found between the species at the level of karyotype composition, and distribution of repetitive DNA classes–either specific ones or the uncharacterized repetitive DNA fraction revealed by CGH. Taking an important role in the initial steps of sex chromosome differentiation, in post-zygotic reproductive isolation, or by contributing to local adaptation in certain populations, chromosomal inversions can suppress the recombination, especially around the rearrangement breakpoints [69,70,71,72,73,74,75,76,77]. In addition, repetitive DNAs may also play an important role in promoting biodiversity, in the differentiation of sex-specific chromosomal regions, and speciation of diverse eukaryotic organisms including fishes [78,79,80,81]. As evidenced in our present study, the differences in karyotype organization and number and distribution of rDNA sites are instrumental as chromosomal markers for the determination of *Harttia* species with 2n = 54 chromosomes. The fixation of such karyotype differences might be facilitated by vicariant events after the Serra do Cachimbo uplifting [36]. This event seems to have played a significant role in the diversification of *Harttia* species, as pointed out by the position of the clades in the phylogenetic reconstruction of this genus. Species from Xingu and Tocantins-Araguaia basins, which have at least two different sex chromosome systems [36], form a sister clade to the branch which encompasses species from the Tapajós basin [57]. One member of the latter group investigated herein, *H. dissidens*, does not present a cytologically detectable sex chromosome system. Additionally, *H. punctata*, a species from the Tocantins-Araguaia basin which possesses an X_1_X_1_X_2_X_2_/X_1_X_2_Y multiple sex chromosome system, corresponds to the sister group of all those above-mentioned species in their phylogenetic reconstruction ([52], Figure 7). 

### 4.2. Sex Chromosomes in Harttia species

At least nine distinct sex chromosome systems are described among fishes, so far known to be distributed in approx. 1% of teleost species (440 cases according to [83]). Many of these systems present only subtle genetic differentiation, which is not detectable by standard cytogenetic methods [19,83]. 

Until now, *Harttia* has presented three distinct male-heterogametic sex chromosome systems: (i) X_1_X_1_X_2_X_2_/X_1_X_2_Y found in *H. punctata* [34], *H. duriventris* and *H. villasboas* [36], (ii) XX/XY_1_Y_2_ found in *H. carvalhoi* [33,34], *H. intermontana* and *Harttia* sp. 1 [35], and (iii) the putative XX/XY sex chromosome system at an early stage of differentiation proposed for *H. rondoni* [36]. Consequently, *Harttia* can be recognized as a taxon with high karyotype dynamics, which is prone to formation of multiple sex chromosome systems. Up to now, 75 cases of multiple sex chromosomes with predicted 60 independent origins have been reviewed by Sember et al. [83] and another two systems have been concomitantly described in *Harttia* [35]. Together with *Nothobranchius*, *Harttia* is the genus with highest number (N = 6) of multiple sex chromosomes (reviewed in ref. [83]). 

Interestingly, although largely different from many viewpoints, both mentioned teleost taxa (*Harttia* and *Nothobranchius*) share the evolution in small allopatric populations with restricted gene flow and propensity to fusions and fissions. It is becoming increasingly apparent that Robertsonian rearrangements might have a significant effect on the nuclear organization of chromosomes along with gene evolution and expression (e.g., [84]). Although present in seven out of the 14 karyotyped species, differentiated sex chromosomes are still intensely discussed concerning the ancestral karyotype of *Harttia*. For this, two main scenarios have been already proposed [36]. The first one—“the proto-XY hypothesis”— considers both X and Y chromosomes to differ only by amplification or contraction of tandem repeats, thus representing an early stage of sex chromosome differentiation. Following this presumption, a proto-XY sex chromosome system, like the one found in *H. rondoni*, could be present in the ancestral karyotype and, consequently, would have originated the X_1_X_2_Y sex chromosome system found in *H. duriventris*, *H. punctata*, and *H. villasboas* via centric fissions [36]. A possible second hypothesis, named “the neo-XY system”, considers the fusions of ancestral X and Y chromosomes with an autosome pair, thus leading to the formation of neo-XY sex chromosomes accompanied by the reduction of 2n [36]. A third hypothesis partially overlaps with the second one and it can be considered for the southeast Brazilian clade of *Harttia*, which does not present any proto-sex, or heteromorphic XY sex chromosomes identified so far. In this case, similar CGH patterns and the phylogenetic relationships between *H. torrenticola* (without differentiated sex chromosomes) and *H. carvalhoi* (with an XY_1_Y_2_ sex system), point to a Robertsonian fusion originating the X-chromosome of *H. carvalhoi*, *H. intermontana*, and *Harttia* sp. 1, as well as the first chromosome pair of *H. torrenticola* [35]. Such hypotheses, especially the first two of them, were proposed assuming the scenario in which all species from the northern Brazilian region present differentiated sex chromosomes in their karyotypes. However, this is not the case, as the present CGH experiments confirmed that *H. dissidens* and *H. guianensis* do not possess differentiated sex chromosomes, at least not at the stage which could be detected by CGH [47,85]. These data suggest rather early steps of differentiation in the *Harttia* species with XX/XY_1_X_2_ sex chromosome system when compared to the karyotypes with homomorphic sex chromosomes in the clade from northern Brazilian region. 

Recent genomic studies continue to reveal homomorphic sex chromosome systems among fishes, thus pointing to a potentially gross underestimation regarding the counts of fish sex chromosomes cases by previous cytogenetic reports [86,87]. This might especially apply to the *Harttia* genus, where several cryptic and undescribed species occur, in addition to the scarcity of cytogenetic (and a complete absence of genomic) data, especially for species from both northern Brazil and Guyana shield. Our CGH experiments utilizing male and female genomic probes to be compared on the male chromosome background in *Harttia* sp. 3, revealed a specific pericentromeric region in one metacentric pair (pair 2) highlighted more (but not exclusively) by the male genomic probe. This observation might point to a nascent region of suppressed recombination on homomorphic sex chromosomes [47], bearing in mind that specific repetitive DNA accumulations might happen on corresponding regions of both sex chromosomes [88]; however, we cannot entirely discard the possibility of intraspecific variability in the copy number and distribution of certain repetitive DNAs. 

## Figures and Tables

**Table 1:** Geographic coordinates of the collection sites and the number of specimens per species analyzed in this study. The number codes placed before the species names correspond to the numbering system in Figure 1.

Species	Locality	n
3-*Harttia dissidens*	Upper section of Grim waterfall, Tambor stream, Rurópolis–PA, Brazil (4°5′37.8″ S 55°0′30.2″ W)	25♂, 07♀
6-*Harttia guianensis*	Paraíso stream (formerly known as Inferno stream), Alenquer–PA, Brazil (1°29′02.2″ S 54°50′31.2″ W)	10♂, 06♀
17-*Harttia* sp. 3	Rio do Peixe, Cachoeira da Serra, Altamira–PA (08°39′20.7″ S 55°09′24.1″ W)	15♂,11♀

## Data Availability

The data presented in this study are available on request from the corresponding author.
